# The complete chloroplast genome of *Euphorbia neriifolia* L. (Euphorbiaceae)

**DOI:** 10.1080/23802359.2025.2569564

**Published:** 2025-10-06

**Authors:** Huang Zhu, Qingjie Li

**Affiliations:** School of Preclinical Medicine, Chengdu University, Chengdu, China

**Keywords:** Chloroplast genome, Euphorbiaceae, *Euphorbia neriifolia*, phylogenetic analyses

## Abstract

*Euphorbia neriifolia* L. is a medicinal and ornamental species widely distributed in India and China, yet its complete chloroplast genome has not been reported. Here, we present its first complete plastome (164,435 bp; GC: 34.97%), featuring a quadripartite structure with a large single-copy (92,653 bp), a small single-copy (18,282 bp), and two inverted repeat (26,750 bp each) regions. The genome encodes 124 genes, including 79 protein-coding genes, 37 tRNAs, and eight rRNAs. Phylogenetic analysis revealed a close relationship with *E. royleana*. This genome provides a fundamental resource for phylogenetic studies and further genetic research in *Euphorbia*.

## 1. Introduction

*Euphorbia neriifolia* L. is a species of succulent shrubby small tree plant of the genus *Euphorbia* in the family Euphorbiaceae, native to India and widespread in many provinces of China. This species can be used as a hedge or grown in greenhouses as an ornamental. Its stems and leaves can also be crushed and applied externally to treat carbuncles and scabies. *E. neriifolia* has a long history of medicinal use in India and the Dai region of China. It has the effects of clearing heat and removing toxins, moistening the bowels and laxative, relieving coughs and calming asthma, etc. It is used in the treatment of emphysema, lung diseases, bronchitis, gastroenteritis, constipation, renal edema, and many other diseases (Mali and Panchal 2017).

The chloroplast genome of angiosperms has been demonstrated to offer a robust instrument for the estimation of species trees, the analysis of population genetics, the function of chloroplast genes and the metabolism of chloroplasts (Dobrogojski et al. 2020). Furthermore, it facilitates the investigation of species adaptation to extreme environments (Daniell et al. 2016; Wang et al. 2024). However, to date, the complete chloroplast genome of *E. neriifolia* has not been reported, which hinders a more profound comprehension of its genomic characterization and evolutionary history.

In this study, the chloroplast genome of *E. neriifolia* was first constructed, and its phylogenetic position in *Euphorbia* sect. Euphorbia was confirmed. As the inaugural plastome resource for this species, it will facilitate valuable contributions to research in the field of comparative genomics and genetics within the genus *Euphorbia* and its family, Euphorbiaceae.

## 2. Materials and methods

### 2.1. Plant materials and sampling

**Figure 1.**
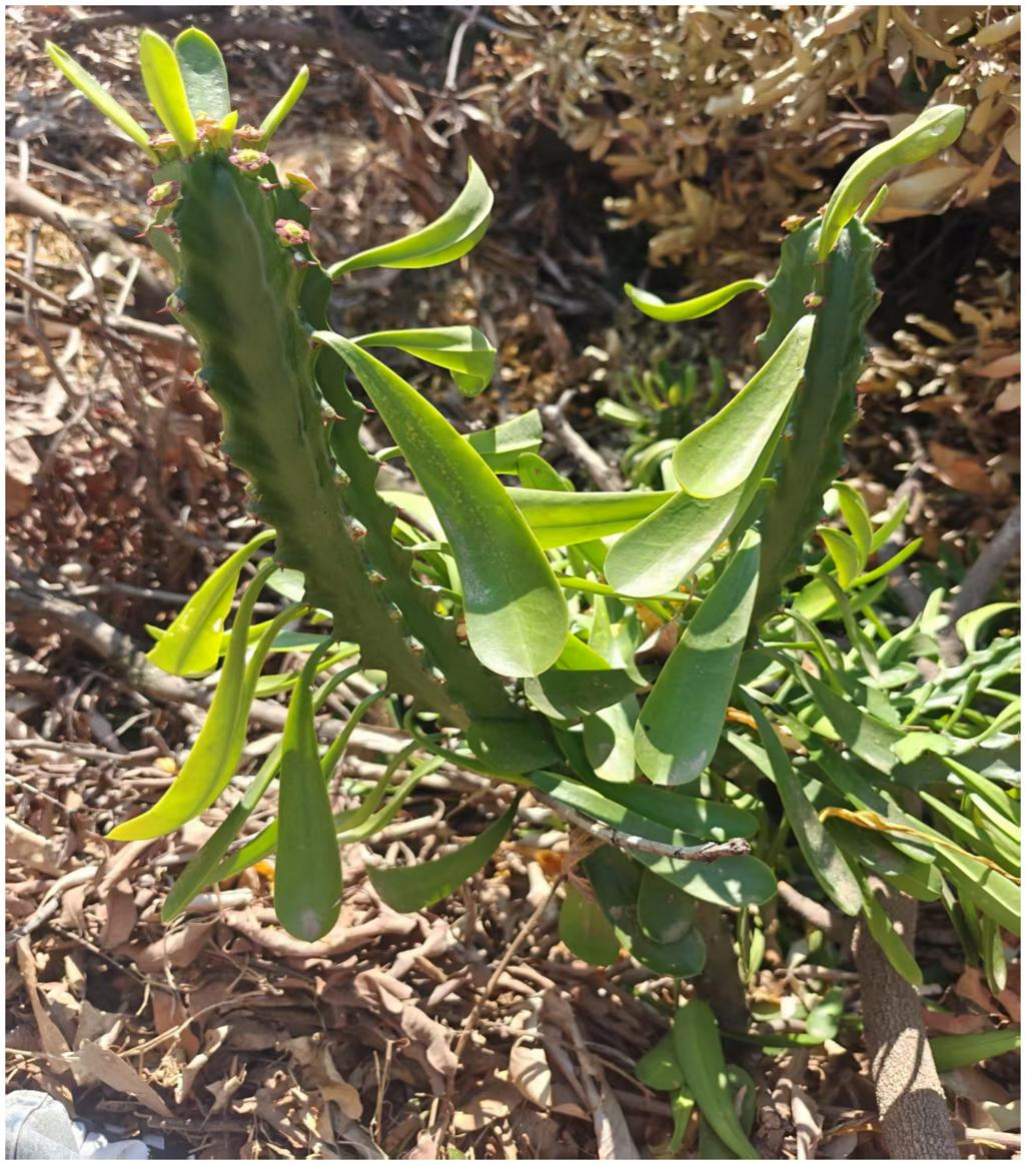
Morphology of *E. neriifolia*. Photograph of a specimen showing its thick, green, succulent stems with faint longitudinal ridges arranged in a spiral, and obovate to spatulate leaves clustered at the upper stem nodes, which are characteristic of the species. Image credit: Qingjie Li (qingjieli2005@163.com).

Fresh and mature leaves from an adult *E. neriifolia* specimen were collected in Panzhihua City, Sichuan Province, China (26.4774°N, 101.7519°E) (Figure 1), in strict accordance with the Regulations of the People’s Republic of China on the Protection of Wild Plants. A specimen was deposited at the museum of Chengdu University of Traditional Chinese Medicine (contact Huang Zhu, zhuhuang@cdu.edu.cn, voucher number: CDU20250216006).

### 2.2. Genome sequencing, assembly, and annotation

For whole-genome DNA sequencing, a modified CTAB method was employed for the extraction of total genomic DNA (Doyle and Doyle 1987). The construction of paired-end Illumina libraries, characterized by an insertion size of 300 base pairs, was undertaken in accordance with the manufacturer’s instructions. The subsequent process of sequencing was performed on an Illumina NovaSeq X Plus platform (Illumina, San Diego, CA), resulting in a total of 6.40 Gb Illumina reads. Subsequently, the chloroplast genome of *E. neriifolia* was *de novo* assembled by GetOrganelle v1.7.7.1 (Jin et al. 2020) using the following parameters: ‘-F embplant_pt’ and ‘-k 21,45,65,85,105’, and the plastome assembly was annotated using Plann v1.1 (Huang and Cronk 2015) with the *E. maculata* chloroplast genome (GenBank accession number: OQ184027) annotation as the reference. The obvious annotation errors were corrected by Geneious (Kearse et al. 2012).

### 2.3. Phylogenetic analysis

We performed phylogenetic analysis among *E. neriifolia* and four other species from *Euphorbia* sect. Euphorbia, including *E. poissonii* (NC_068487), *E. ampliphylla* (NC_068488), *E. drupifera* (NC_062829), and *E. royleana* (NC_073015), using *E. torrei* (MT395013) and *E. ritchiei* (NC_068459) as outgroups, which are phylogenetically distant congeners outside of sect. Euphorbia. The coding sequences of PCGs present in all the seven chloroplast genomes of *Euphorbia* species were aligned by MAFFT-LINSI v7.313 (Katoh and Standley 2013). The resulting alignments were then subjected to a process of concatenation, resulting in the construction of a supermatrix. This was followed by the generation of a maximum-likelihood phylogenetic tree using the program RAxML (versions 8.2.11) (Stamatakis 2006) under the GTRGAMMA model with 1000 bootstrap replicates. Furthermore, a comparison was made of the seven chloroplast genomes of *Euphorbia* species utilizing the DnaSP v5.10 software (Librado and Rozas 2009), with a window size and step size set at 800 and 200 bp, respectively.

## 3. Results

**Figure 2.**
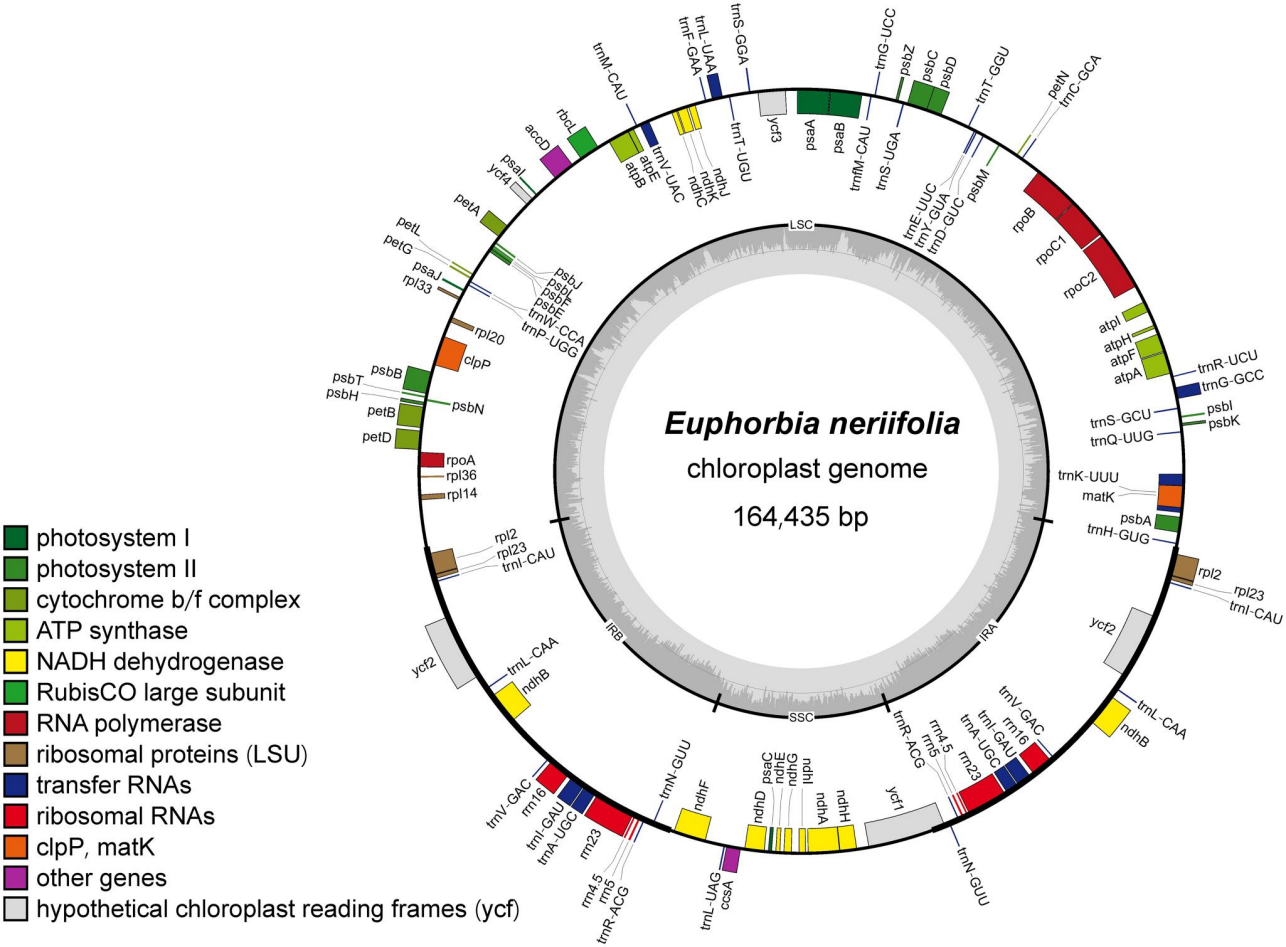
Physical map of *E. neriifolia* chloroplast genome. The genes shown on the outside of the circle are transcribed counterclockwise, while those on the inside are transcribed clockwise. The colored arcs indicate genes belonging to different functional groups (see the legend for color codes). The inner gray circle delineates the large single-copy (LSC), small single-copy (SSC), and inverted repeat (IRa and IRb) regions. The inner gray histogram represents the GC content; higher peaks correspond to regions of elevated GC content.

The genome sequence of *E. neriifolia* yielded in a total of 6.40 Gb of Illumina short-read data. The chloroplast genome sequence of *E. neriifolia* (GenBank accession number: PV440225) revealed a circular structure spanning 164,435 bp. This structure contains two IR regions of 26,750 bp, separated by a LSC region of 92,653 bp and a SSC region of 18,282 bp (Figure 2 and Supplementary Table 1). The mean coverage depth of *E. neriifolia* chloroplast genomes was determined to be 2414× (Supplementary Figure 1).

The chloroplast genome in question contained 124 genes, including 79 PCGs, 37 tRNA genes, and eight rRNA genes (Supplementary Table 1). Of these, 10 PCGs and eight tRNA genes had a single intron, and three PCGs (*rps12*, *ycf3*, and *clpP*) had two introns. The structure of the 16 cis-spliced genes is shown in Supplementary Figure 2, and the structure of trans-spliced gene *rps12* was verified and is illustrated in Supplementary Figure 3. The overall GC content was determined to be 34.97%, while the GC content for the first, second, and third codons of PCGs was found to be 45.27%, 37.84%, and 28.10%, respectively (Supplementary Table 1). The 79 PCGs genes contained a total of 23,941 codons, and leucine and cystine ranking as the most and least frequently utilized amino acids, respectively, with a frequency of 10.63% and 1.17%.

**Figure 3.**
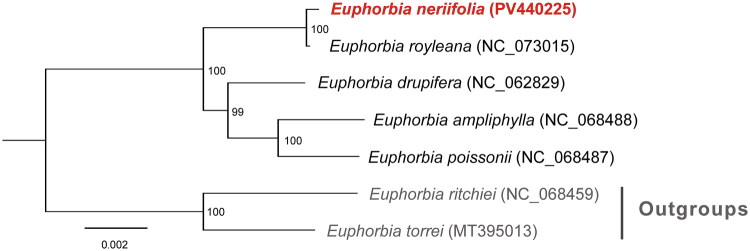
A maximum-likelihood tree showing the phylogenetic relationships between *E. neriifolia* and six other *Euphorbia* species. Bootstrap values were denoted on each internal node. The study utilized the following chloroplast genome sequences from the NCBI database, which were publicly available but not yet formally published: *E. poissonii* (NC_068487), *E. ampliphylla* (NC_068488), *E. drupifera* (NC_062829), *E. royleana* (NC_073015), *E. torrei* (MT395013, outgroup), and *E. ritchiei* (NC_068459, outgroup). The species analyzed in this study (*E. neriifolia*) is highlighted in red and bold in the phylogenetic tree.

A comprehensive analysis of 71 PCGs, encompassing all the plastomes of *E. neriifolia* and six additional *Euphorbia* species, was conducted to elucidate its evolutionary relationships. The results of this analysis, as illustrated in Figure 3, indicated that *E. neriifolia* was closely related to *E. royleana*, a relationship that received 100% bootstrap support.

In addition, comparison of seven chloroplast genomes (Supplementary Figure 4) identified several highly variable regions with high nucleotide diversity values (>0.02), including the genic regions of nine PCGs (*psbA*, *rpl33*, *rps18*, *rpl16*, *rps3*, *rpl22*, *ndhF*, *ccsA*, and *ycf1*) and three tRNA genes (*trnS-GCU*, *trnE-UUC*, and *trnT-GGU*). These regions have the potential to function as molecular markers during the course of phylogenetic analyses of *Euphorbia* species.

## 4. Discussion and conclusions

The complete chloroplast genome of *E. neriifolia* was assembled and annotated for the first time, revealing a genome of 164,435 bp, 124 genes, and an overall GC content of 34.97%. These features are consistent with previously reported *Euphorbia* plastomes, which generally maintain a conserved quadripartite structure and similar gene content (Wei et al. 2021; Lee et al. 2024).

The phylogenetic analysis based on 71 protein-coding genes placed *E. neriifolia* in close proximity to *E. royleana*, both belonging to *Euphorbia* subgenus *Euphorbia*. Morphologically, these two species share the unusual trait within the subgenus of producing relatively large, persistent leaves clustered at the tips of branches (Dorsey et al. 2013). By contrast, *E. poissonii*, *E. ampliphylla*, and *E. drupifera* typically have smaller or deciduous foliage and distinct branching habits. These consistent morphological distinctions support the close affinity between *E. neriifolia* and *E. royleana* inferred from the plastome data.

Furthermore, the identification of hypervariable regions such as *ycf1*, *ndhF*, and *psbA* aligns with previous *Euphorbia* plastome studies, underscoring their value as molecular markers (Wei et al. 2021). *ycf1* is among the most variable and reliable plastid barcoding loci across land plants (Dong et al. 2015), and together with *ndhF* has shown strong discriminatory power at lower taxonomic levels (Amar 2020), supporting their utility for future *Euphorbia* systematics.

Overall, this work not only fills a gap in the plastome resources of *E. neriifolia* but also provides a foundation for future taxonomic, evolutionary, and ecological studies. Expanding taxon sampling, integrating nuclear genomic data, and conducting population-level analyses will be critical for elucidating the evolutionary history and adaptive significance of plastome variation in *Euphorbia* and across Euphorbiaceae, with potential applications in species conservation and medicinal plant development.

## Supplementary Material

Supplementary material.docx

## Data Availability

The chloroplast genome assembly and raw sequencing data in this study are available in NCBI (https://www.ncbi.nlm.nih.gov/) under the accession numbers of PV440225 and SRR32948832 (BioSample: SAMN47749030; BioProject: PRJNA1245319).
