# Supplementary material for: The complete chloroplast genome of Euphorbia neriifolia L. (Euphorbiaceae)
Source: Mitochondrial DNA B Resour. 2025 Oct 6;10(11):1012–5. doi: 10.1080/23802359.2025.2569564 (PMC12502097; doi:10.1080/23802359.2025.2569564)
Supplement: Supplementary material.docx [file TMDN_A_2569564_SM5036.docx]

**The complete chloroplast genome of *Euphorbia neriifolia* L. (****Euphorbiaceae)**

Huang Zhu^a*^, Qingjie Li^a^

^a^ School of Preclinical Medicine, Chengdu University, Chengdu, China

*^*^***CONTACT** Huang Zhu; zhuhuang@cdu.edu.cn; School of Preclinical Medicine, Chengdu University, Chengdu, China

**ORCID** Huang Zhu; https://orcid.org/0009-0005-3706-2787


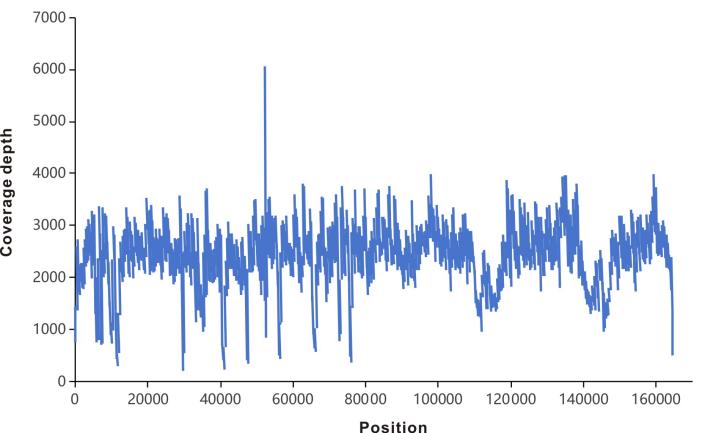


**Supplementary Figure 1**. Coverage depth distribution of the *E. neriifolia* chloroplast genome. The sequencing depth ranged from 200× to 6055×, with an average depth of 2414×.


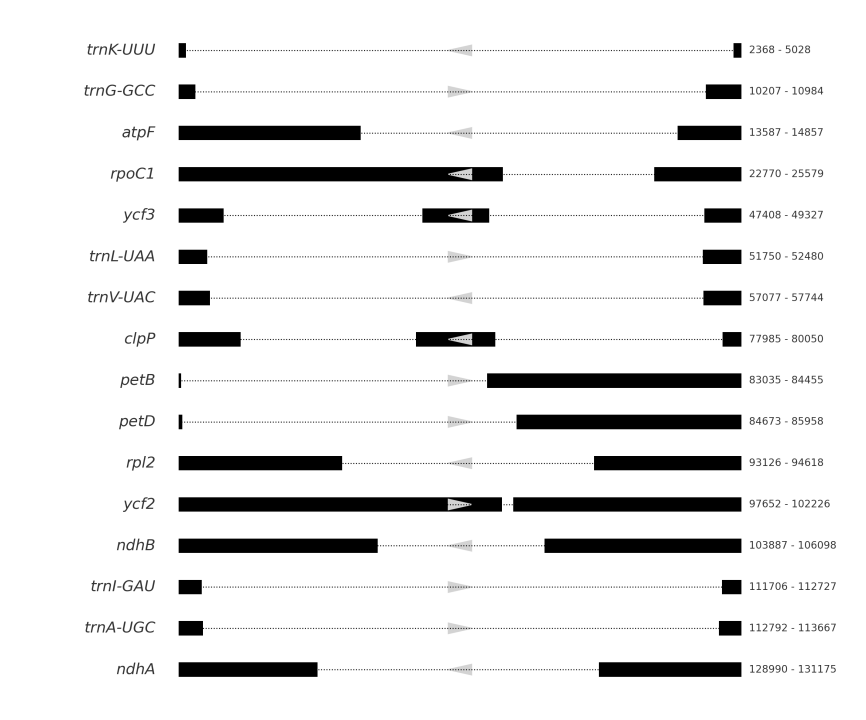


**Supplementary Figure 2**. Exon-intron structures of 16 cis-spliced genes in the *E. neriifolia* chloroplast genome. Black boxes represent exons, and dotted lines indicate introns. Numbers to the right of each row indicate the start and end positions of the corresponding gene in the chloroplast genome. Grey arrows indicate transcriptional direction.


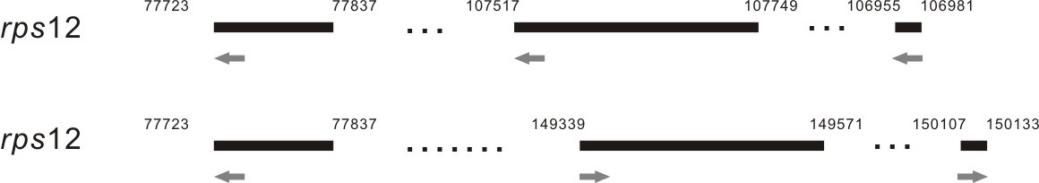


**Supplementary Figure 3**. Structure of the trans-splicing gene *rps12* in the *E. neriifolia* chloroplast genome.


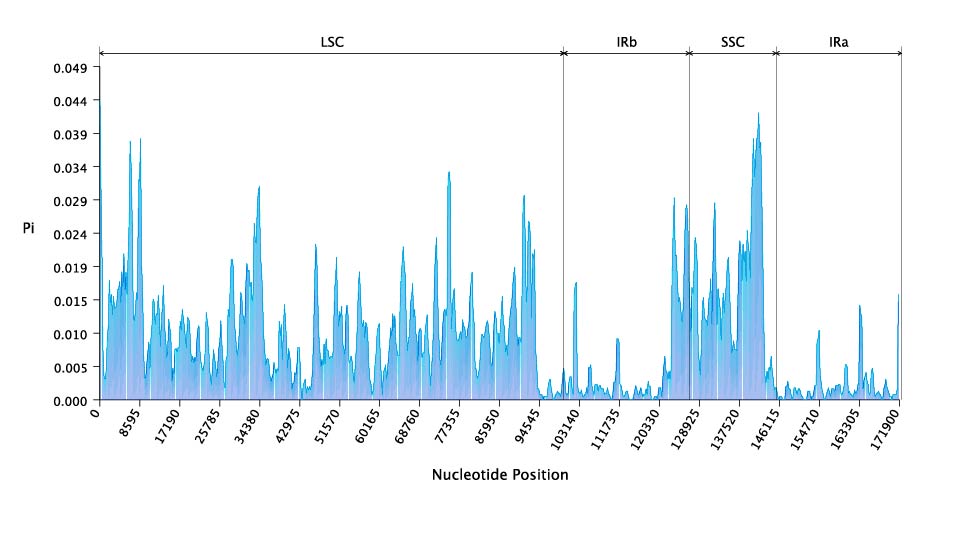


**Supplementary Figure 4**. Results of nucleotide diversity analyses for seven other *Euphorbia* species

**Supplementary Table 1** Assembly and annotation statistics of *E. neriifolia* chloroplast genome.

| **Features** | **Values** |
| --- | --- |
| Total sequenced bases (bp) | 6,403,676,700 |
| Coverage depth (×) | 2,414 |
| Total size (bp) | 164,435 |
| LSC size (bp) | 92,653 |
| SSC size (bp) | 18,282 |
| IR size (bp) | 26,750 |
| Overall GC content (%) | 34.97 |
| GC content in the first codons (%) | 45.27 |
| GC content in the second codons (%) | 37.84 |
| GC content in the third codons (%) | 28.10 |
| Number of genes | 124 |
| Number of protein-coding genes | 79 |
| Number of tRNAs | 37 |
| Number of rRNAs | 8 |
